# Diagnostic Performance of Advanced Diffusion MRI Parameters Versus Conventional Diffusion-Weighted Imaging (DWI) for Detecting Lymph Node Metastasis in Colorectal Cancer: A Systematic Review and Meta-Analysis

**DOI:** 10.7759/cureus.99930

**Published:** 2025-12-23

**Authors:** Salim Ur Rahman, Moomna Khalid

**Affiliations:** 1 Acute Medicine, Countess of Chester Hospital, Chester, GBR; 2 Radiology, Lady Reading Hospital, Peshawar, PAK

**Keywords:** advanced diffusion mri, colorectal cancer, diagnostic accuracy, ivim, lymph node metastasis

## Abstract

Accurate preoperative identification of lymph node metastasis (LNM) in colorectal cancer is essential for optimal staging and treatment planning. Conventional diffusion-weighted imaging (DWI) is limited by overlap in apparent diffusion coefficient (ADC) values between benign and malignant lymph nodes. Advanced diffusion MRI techniques, including intravoxel incoherent motion (IVIM) parameters (diffusion coefficient (D), pseudodiffusion coefficient (D*), and perfusion fraction (f)) and diffusion tensor imaging (DTI) metrics such as fractional anisotropy (FA), axial diffusivity (AD), mean diffusivity (MD), and radial diffusivity (RD), may improve diagnostic accuracy. This systematic review and meta-analysis evaluated the diagnostic performance of these advanced diffusion parameters compared with conventional DWI. A comprehensive search of PubMed, Google Scholar, ScienceDirect, and the Cochrane Library identified studies assessing diffusion MRI for LNM detection with extractable true-positive, false-positive, false-negative, and true-negative data. Five studies met the inclusion criteria. Risk ratios (RRs) were pooled using RevMan (The Cochrane Collaboration, London, United Kingdom), and bivariate summary receiver operating characteristic (SROC) analysis was performed in R (R Development Core Team, Vienna, Austria). Study quality was assessed using QUADAS-2 (Quality Assessment of Diagnostic Accuracy Studies-2). Five studies involving 239 patients and 358 lymph nodes evaluated IVIM, DTI, ADC, and multiparametric models. IVIM-derived D showed significantly superior performance compared with ADC (RR=9.11, 95% CI: 4.86-17.09; I²=0%), while D* also demonstrated strong discriminatory ability (RR=3.12, 95% CI: 2.10-4.65; I²=0%). SROC analysis revealed high diagnostic accuracy for IVIM D (area under the curve (AUC)≈0.93), moderate accuracy for D* (AUC≈0.82), and the highest accuracy for combined parameters (AUC≈0.95). DTI metrics, particularly FA and AD, consistently showed high sensitivity (88-100%) and specificity (85-100%). Overall risk of bias was low. Advanced diffusion MRI parameters outperform conventional DWI for detecting LNM in colorectal cancer, with multiparametric approaches offering the greatest diagnostic value.

## Introduction and background

Colorectal cancer remains a major global health burden, accounting for more than 1.9 million new cases each year and ranking among the leading causes of cancer-related mortality worldwide [1]. Early and accurate staging is critical because treatment pathways differ substantially based on local tumor extent and nodal involvement. In particular, lymph node metastasis (LNM) is one of the strongest predictors of recurrence risk and overall prognosis, directly informing the need for neoadjuvant chemoradiation and influencing decisions regarding total mesorectal excision, sphincter preservation, and organ-saving strategies [2]. However, despite its clinical importance, reliably identifying metastatic lymph nodes preoperatively remains challenging. Conventional MRI primarily relies on morphologic criteria such as size, border irregularity, and internal signal heterogeneity, yet these features often overlap between benign and malignant nodes [3]. Reactive or inflammatory nodes may enlarge, while metastatic nodes may remain small, leading to considerable diagnostic uncertainty [4]. As a result, morphological assessment alone frequently yields suboptimal diagnostic performance.

Diffusion-weighted imaging (DWI) and its quantitative parameter, the apparent diffusion coefficient (ADC), have been widely adopted to enhance tissue characterization in rectal and colorectal cancer [5]. ADC provides insight into tissue cellularity and microscopic water motion, offering a functional dimension beyond morphology. Nevertheless, ADC-based assessment of lymph nodes is limited by significant overlap between benign and metastatic nodes, dependency on b-value selection, and sensitivity to perfusion contamination and image noise [3-6]. These limitations contribute to inconsistent diagnostic accuracy across studies and prevent ADC from serving as a reliable standalone biomarker for nodal staging [6-8]. Moreover, similar constraints of ADC have been demonstrated across other organ systems, including liver and head-and-neck imaging, highlighting that these limitations arise from the mono-exponential diffusion model itself rather than from disease-specific factors [7,8].

To address the limitations of ADC, several advanced diffusion MRI models, such as intravoxel incoherent motion (IVIM), diffusion tensor imaging (DTI), and physiologic contrasts including amide proton transfer (APT) imaging and T1 mapping, have been developed to characterize tissue microstructure and perfusion more accurately. IVIM separates diffusion from perfusion effects [9,10], DTI assesses directional water diffusion [11], and APT/T1 techniques provide additional biochemical and relaxation-based contrasts [12]. Although APT imaging and T1 mapping remain relatively new in the context of colorectal cancer, the early evidence supporting their diagnostic potential justifies their inclusion as emerging complementary techniques.

Despite promising findings, prior studies vary considerably in methodology and diagnostic thresholds, and the available evidence remains fragmented. Importantly, no prior synthesis has directly compared IVIM-, DTI-, and physiologic MRI-derived parameters with conventional ADC for detecting colorectal LNM. Therefore, this systematic review and meta-analysis aim to evaluate and compare the diagnostic accuracy of these advanced diffusion MRI techniques. By generating pooled diagnostic estimates and summary receiver operating characteristic (SROC) curves, this study clarifies the relative performance of advanced diffusion parameters and their potential added value over ADC, thereby informing future directions for preoperative nodal staging.

## Review

Materials and methods

A systematic search was undertaken across PubMed, the Cochrane Library, ScienceDirect, and Google Scholar to identify studies evaluating advanced diffusion MRI techniques for detecting LNM in colorectal cancer. Searches were conducted without publication date limits, and the most recent search update was completed on October 20, 2025. The search strategy combined terms related to colorectal cancer and diffusion MRI, including “colorectal cancer,” “rectal cancer,” “lymph node metastasis,” “advanced diffusion MRI,” “IVIM,” “diffusion tensor imaging,” “DTI,” “APT imaging,” “T1 mapping,” “multi-b value,” “ADC,” and “diagnostic performance.” Reference lists of included articles were reviewed to identify any additional relevant publications. Non-peer-reviewed sources such as dissertations, unpublished work, conference abstracts, and trial registries were not systematically explored, as the focus was placed on indexed journal literature to ensure methodological consistency and data reliability. The core search terms were applied consistently across all databases, and no additional filters or date limits were used beyond restricting results to English-language peer-reviewed studies, ensuring that the strategy remains reproducible.

Eligibility Criteria

Eligible studies were required to include adult participants (aged 18 years or older) with histologically confirmed colorectal cancer who underwent preoperative MRI for assessment of lymph node status. To be considered for inclusion, studies needed to evaluate at least one advanced diffusion MRI parameter, such as IVIM-derived metrics (diffusion coefficient (D), pseudodiffusion coefficient (D*), and perfusion fraction (f)), DTI-based measures (fractional anisotropy (FA), axial diffusivity (AD), mean diffusivity (MD), or radial diffusivity (RD)), or physiologic contrasts including APT imaging or T1 mapping, and directly compare these parameters with conventional DWI using the ADC. Only peer-reviewed, English-language publications that provided sufficient diagnostic accuracy data to derive or reconstruct true-positive (TP), false-positive (FP), false-negative (FN), and true-negative (TN) values were eligible. For studies enrolling mixed cancer populations, only colorectal cancer-specific lymph node data were extracted to maintain consistency with the predefined eligibility criteria.

Studies were excluded if they lacked quantitative diagnostic accuracy data for LNM, assessed only conventional DWI without incorporating advanced diffusion techniques, focused on diseases other than colorectal cancer, or did not use histopathology as the reference standard. Non-original publications, including reviews, commentaries, case reports, editorials, and conference abstracts, were also excluded. For studies that enrolled mixed patient populations, inclusion was permitted only when colorectal cancer-specific data were reported separately, in which case only the relevant subgroup data were extracted for analysis (Table 1).

**Table 1:** Eligibility criteria for study inclusion IVIM, intravoxel incoherent motion; DTI, diffusion tensor imaging; DWI, diffusion-weighted imaging; ADC, apparent diffusion coefficient; TP, true positives; FP, false positives; FN, false negatives; TN, true negatives; LNM, lymph node metastasis; APT, amide proton transfer; D, diffusion coefficient; D*, pseudodiffusion coefficient; f, perfusion fraction; FA, fractional anisotropy; AD, axial diffusivity; MD, mean diffusivity; RD, radial diffusivity

Domain	Inclusion criteria	Exclusion criteria
Population	Adults (≥18 years) with histologically confirmed colorectal cancer undergoing preoperative MRI for lymph node evaluation	Non-colorectal cancers; mixed populations without separable colorectal cancer data; pediatric populations
Index tests	Advanced diffusion MRI techniques including IVIM (D, D*, f), DTI (FA, AD, MD, RD), APT imaging, or T1-mapping	Studies evaluating only conventional DWI/ADC without advanced diffusion parameters
Comparator	Conventional mono-exponential DWI using ADC	Studies lacking a comparator or using non-DWI comparators alone
Outcomes	Diagnostic accuracy for LNM with extractable or reconstructable TP, FP, FN, TN values	Studies reporting qualitative findings without diagnostic accuracy data; anatomical/visual outcomes only
Reference standard	Postoperative histopathology for lymph node status	Studies not using histopathology (e.g., clinical follow-up only, radiologic reference alone)
Study design	Prospective or retrospective diagnostic accuracy studies	Reviews, editorials, commentaries, case reports, conference abstracts, theses, animal studies
Language and publication type	English-language, peer-reviewed, indexed publications	Non-English publications; non-peer-reviewed sources; grey literature not providing extractable data

Study Selection

Two reviewers independently screened the retrieved citations by reviewing titles and abstracts to identify studies that met the predefined eligibility criteria. Full texts were obtained for all records considered potentially relevant or when eligibility could not be determined from the abstract alone. Any disagreements regarding study inclusion were resolved through discussion between the two reviewers, with consensus reached in all cases. The selection process adhered to the Preferred Reporting Items for Systematic Reviews and Meta-Analyses for Diagnostic Test Accuracy (PRISMA-DTA) framework [13], and a PRISMA flow diagram was developed to outline the number of studies identified, screened, excluded, and ultimately included in the review.

Data Extraction

A structured data extraction sheet was developed to collect key methodological and diagnostic accuracy information from each study. Extracted variables included author name, year of publication, country, study design, sample size, patient demographics, tumor characteristics, MRI acquisition parameters, advanced diffusion model evaluated, comparator (ADC), and reference standard. Diagnostic accuracy data, TP, FP, FN, and TN, were recorded for each eligible diffusion parameter. When studies did not provide explicit TP, FP, FN, and TN values, these were reconstructed from reported sensitivity, specificity, and sample size, following standard diagnostic accuracy methods. When necessary, sensitivity and specificity values were used to reconstruct 2×2 tables based on reported sample sizes. For studies reporting multiple advanced parameters, data for each parameter were extracted and cataloged separately to allow parameter-specific evaluation.

Quality Assessment

Methodological quality was assessed using the QUADAS-2 (Quality Assessment of Diagnostic Accuracy Studies-2) tool, which evaluates four domains: patient selection, index test, reference standard, and flow and timing [14]. Each domain was rated as having low, high, or unclear risk of bias. Applicability concerns for each domain were also assessed. Both reviewers performed the assessment independently, and discrepancies were resolved through discussion. The overall findings of the risk-of-bias evaluation were presented narratively and visually using a traffic-light plot. We also noted variability in the reporting of time intervals between MRI acquisition and surgical histopathology across studies, and this was considered during the QUADAS-2 bias assessment.

Data Synthesis and Statistical Analysis

Diagnostic accuracy outcomes were synthesized using both qualitative and quantitative approaches. Meta-analysis was conducted for diffusion parameters reported in at least two studies with extractable 2×2 data. Continuity corrections were applied only when necessary to accommodate zero-cell values in reconstructed 2×2 tables. Risk ratios (RRs) with 95% CI were calculated using the Mantel-Haenszel method. RRs were selected as the primary pooled metric because they allow direct comparison of correct classification rates between advanced diffusion models and ADC, and because bivariate sensitivity-specificity pooling was not feasible for parameters reported in only two studies. A fixed-effect model was employed when heterogeneity was low (I²<50%), whereas a random-effects model was planned for substantial heterogeneity. SROC curves were generated using Reitsma’s bivariate model to assess overall diagnostic performance. Parameters reported in fewer than nine studies were summarized descriptively. All statistical analyses were performed using Review Manager (RevMan 5.4, The Cochrane Collaboration, London, United Kingdom) and R (mada package, R Development Core Team, Vienna, Austria).

Results

**Figure 1:**
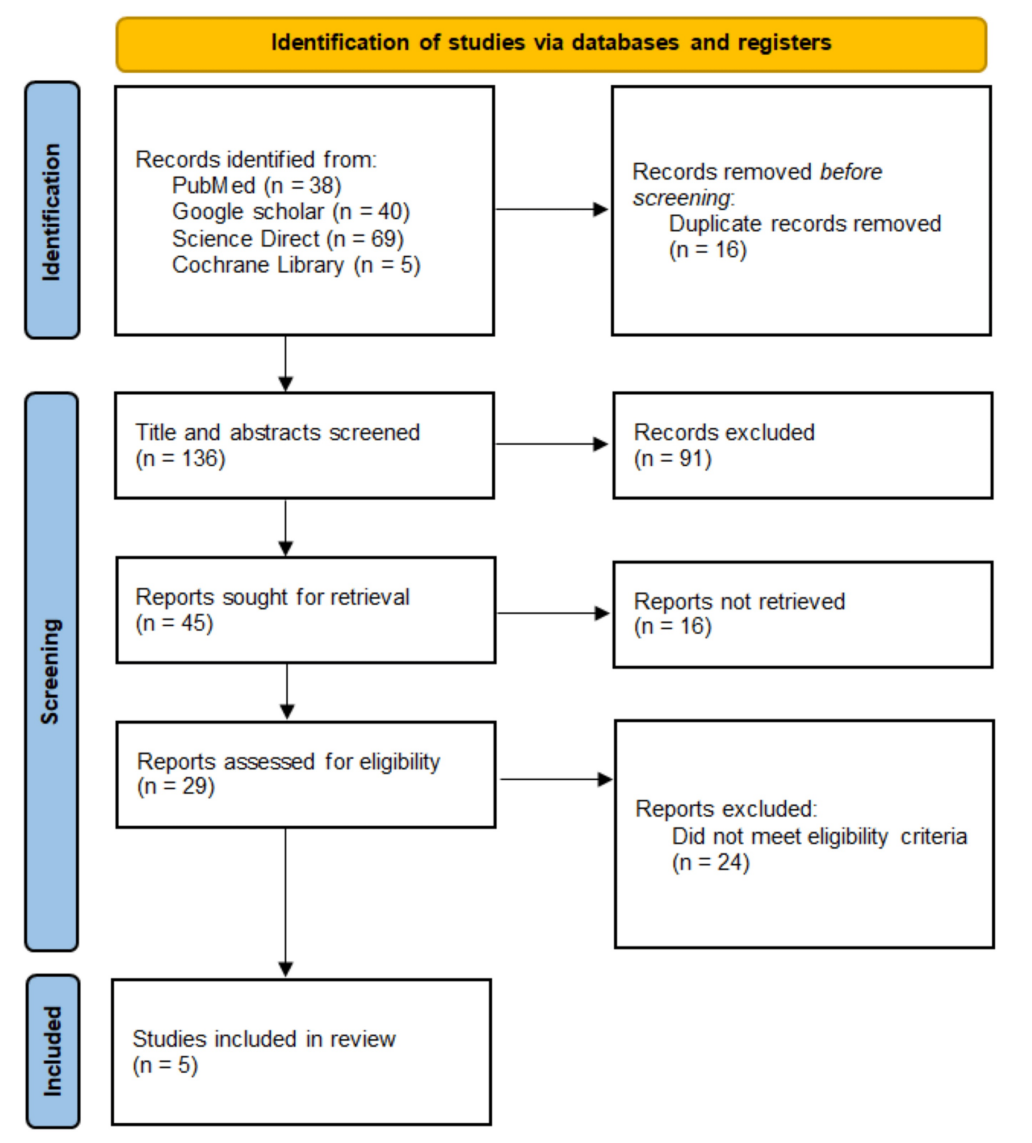
PRISMA flow diagram 2020 PRISMA: Preferred Reporting Items for Systematic Reviews and Meta-Analyses

The database search identified 152 records, of which 16 duplicates were removed. After screening 136 titles and abstracts, 91 articles were excluded for irrelevance. Full texts were sought for 45 studies, but 16 could not be retrieved. Of the remaining 29 articles, 24 were excluded because they did not report advanced diffusion parameters, lacked extractable diagnostic accuracy data, or used unsuitable comparator techniques. Five studies met all eligibility criteria and were included in the final synthesis. The study selection process is summarized in the PRISMA flow diagram (Figure 1).

Study Characteristics

The five included studies, published between 2016 and 2024, collectively enrolled 239 patients who underwent MRI for preoperative lymph node evaluation in colorectal cancer. Across these studies, a total of 358 lymph nodes were assessed histopathologically. The prevalence of LNM varied across individual studies but generally ranged from approximately 40% to 60% based on reported patient- or node-level data. The included studies evaluated a range of advanced diffusion MRI techniques, including IVIM-derived parameters (D, D*, f), DTI metrics (FA, AD, MD, RD), and physiologic contrasts such as amide proton transfer-weighted (APTw) imaging and T1 mapping. One study incorporated a combined multiparametric approach, and two studies evaluated diffusion parameters in the restaging setting after chemoradiation. All studies used postoperative histopathology as the reference standard. Key characteristics of the included studies are summarized in Table 2.

**Table 2:** Summary of the key characteristics of eligible studies included in the review LNM, lymph node metastasis; DTI, diffusion tensor imaging; FA, fractional anisotropy; AD, axial diffusivity; MD, mean diffusivity; RD, radial diffusivity; AUC, area under curve; WDA, well-differentiated adenocarcinoma; MDA, moderately differentiated adenocarcinoma; PDA, poorly-differentiated adenocarcinoma; NET, neuroendocrine tumor; IVIM, intravoxel incoherent motion; APT, amide proton transfer; ADC, apparent diffusion coefficient; D, diffusion coefficient; D*, pseudodiffusion coefficient; f, perfusion fraction; DWI, diffusion-weighted imaging; ROC, receiver-operating characteristic; PPV, positive predictive value; NPV, negative predictive value; pCR, pathological complete response; CRT, chemoradiation therapy; HG, histologic grade; IVIM, intravoxel incoherent motion; APTw, amide proton transfer-weighted

Study (year)	Country	Study design	Sample size	Population details	Mean age (years)	Gender (male/female)	Tumor type	MRI modality and parameters	Quantitative parameters	Diagnostic outcomes reported
Yamada et al., 2020 [15]	Japan	Prospective diagnostic accuracy study	37 patients (LNM+:16; LNM-: 21)	HG: WDA=9, MDA=24, PDA=2, NET=2	64.3±11.6	25/12	Rectal adenocarcinoma	1.5T DTI; b=0,1000; 9 gradient directions	FA, AD, MD, RD	AUC, sensitivity, specificity for HG and LNM prediction
Qiu et al., 2016 [16]	China	Prospective diagnostic accuracy study	68 patients (LNM+:93, LNM-: 67)	Rectal adenocarcinoma	57.7±14.7	41/27	Tubular rectal adenocarcinoma	3.0T IVIM-DWI with 14 b-values (0-2000)	IVIM: D, D*, f; ADC	AUC, sensitivity, specificity, PPV, NPV
Yu et al., 2016 [17]	China	Prospective diagnostic study	32 patients (LNM+:31, LNM-: 28)	Mesorectal lymph nodes	56 (35-73)	12/20	Rectal adenocarcinoma	1.5T IVIM-DWI; 12 b-values (0-800)	Short axis diameter, D, D*, f	AUC, sensitivity, specificity; ROC cutoffs
Wang et al., 2023 [12]	China	Retrospective diagnostic study	23 patients (LNM+:14, LNM-: 9)	LNM prediction	Group A: 65.29; Group B: 69.44	A:10/4; B:6/3	Rectal adenocarcinoma	3.0T APTw + T1 mapping	T1 value, APT (%)	AUC, sensitivity, specificity
Li et al., 2022 [18]	China	Prospective two-center study	79 patients (LNM+:35, LNM-:44)	Also reported pCR and response groups	56.8±9.4	64/15	Locally advanced rectal adenocarcinoma	1.5T IVIM-DWI (11 b-values); T2WI, DWI	D, D*, f; ADC; Δ% change post-CRT	AUC, ORs, HR for outcomes

**Figure 2:**
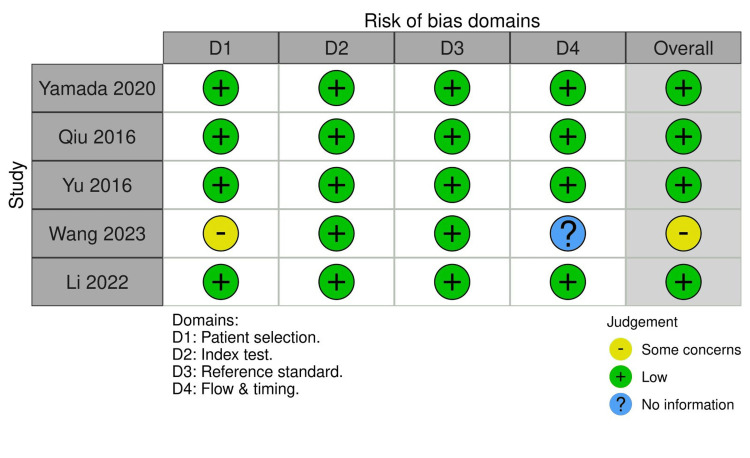
QUADAS-2 risk of bias traffic plot Studies shown are Wang et al. [12], Yamada et al. [15], Qiu et al. [16], Yu et al. [17], and Li et al. [18]. QUADAS-2, Quality Assessment of Diagnostic Accuracy Studies-2

The QUADAS-2 assessment showed generally low risk of bias across the index test and reference standard domains. Most studies used blinded readers and histopathology as the diagnostic benchmark. The main concerns arose in patient selection, where retrospective designs and exclusion of poorly visualized or very small nodes introduced potential bias. Some flow and timing issues were noted for studies omitting nonassessable nodes. Applicability concerns were primarily related to variations in MRI acquisition protocols and diffusion model fitting methods (Figure 2).

Pooled Analysis of IVIM-D (True Diffusion Coefficient)

**Figure 3:**
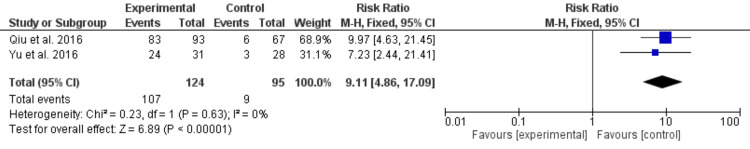
Forest plot of IVIM-D for detecting LNM Forest plot summarizing the pooled diagnostic accuracy (expressed as RR) of the IVIM-D parameter (true diffusion coefficient) for identifying metastatic lymph nodes in colorectal cancer. Studies shown are Qiu et al. [16] and Yu et al. [17]. LNM, lymph node metastasis; RR, risk ratio; IVIM, intravoxel incoherent motion; D, diffusion coefficient

Two studies (Qiu et al. [16] and Yu et al. [17]) reported extractable diagnostic accuracy data for IVIM-derived diffusion coefficient D. Meta-analysis demonstrated that D provided substantially higher accuracy compared with conventional ADC. Using a fixed-effect model, the pooled RR was 9.11 (95% CI: 4.86-17.09), indicating great improvement in the correct identification of metastatic lymph nodes. Heterogeneity was minimal (I²=0%). The forest plot is shown in Figure 3.

Pooled Analysis of IVIM-D*

**Figure 4:**
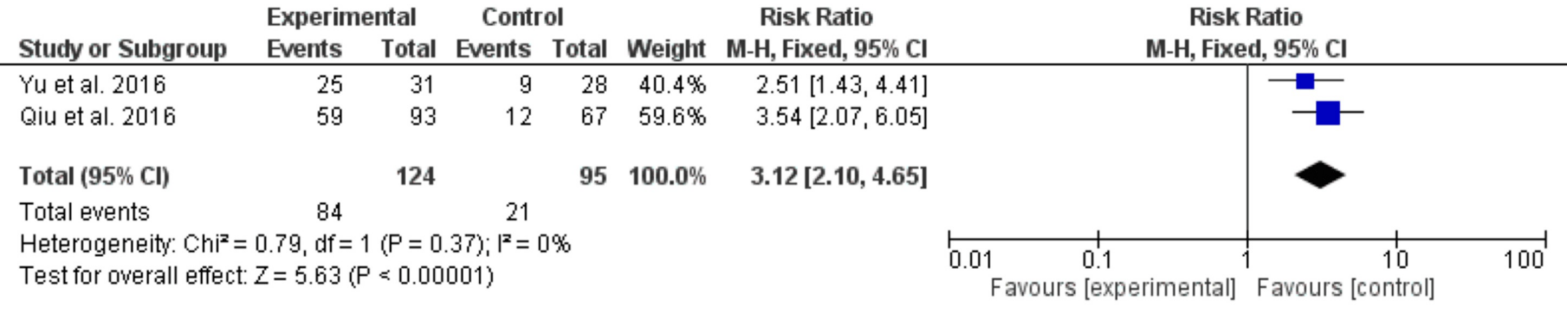
Forest plot of IVIM-D* for detecting LNM Forest plot showing the pooled diagnostic accuracy of the IVIM-D* parameter. Studies shown are Qiu et al. [16] and Yu et al. [17]. LNM, lymph node metastasis; IVIM, intravoxel incoherent motion; D*, pseudodiffusion coefficient

The same two studies [16,17] reported diagnostic accuracy for D*, allowing pooled synthesis. The combined RR for D* was 3.12 (95% CI: 2.10-4.65), reflecting moderate improvement over ADC. Although D* demonstrated discriminatory ability, its magnitude of effect was notably lower than that of D. Heterogeneity remained negligible (I²=0%). The forest plot is presented in Figure 4.

SROC Analysis Using Reitsma’s Bivariate Model

**Figure 5:**
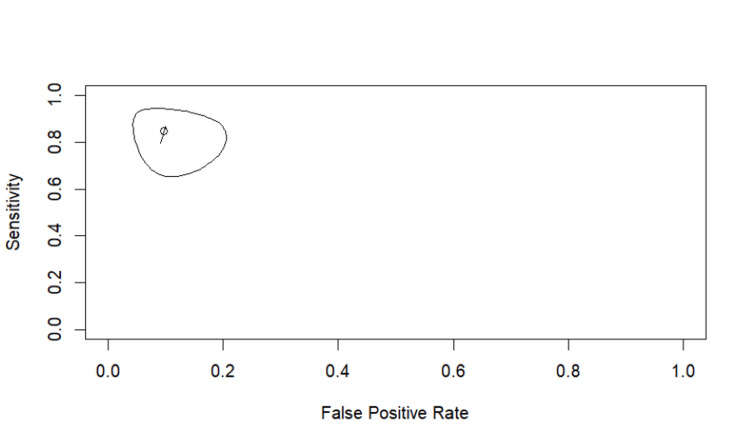
SROC curve for IVIM-D SROC curve generated using Reitsma’s bivariate random-effects model for IVIM-D. Studies shown are Qiu et al. [16] and Yu et al. [17]. IVIM, intravoxel incoherent motion; D, diffusion coefficient; SROC, summary receiver operating characteristic

Using Reitsma's bivariate model, IVIM-D demonstrated high overall diagnostic accuracy, with a summary area under the curve (AUC) of 0.927 and a partial AUC of 0.844. The pooled sensitivity was 0.847 (95% CI: 0.700-0.930), and the pooled specificity was approximately 0.903 based on an FP rate of 0.097 (95% CI: 0.050-0.178). Between-study heterogeneity was minimal across all heterogeneity indices (I²≈0-3%), indicating strong consistency between the two included datasets (Figure 5).

**Figure 6:**
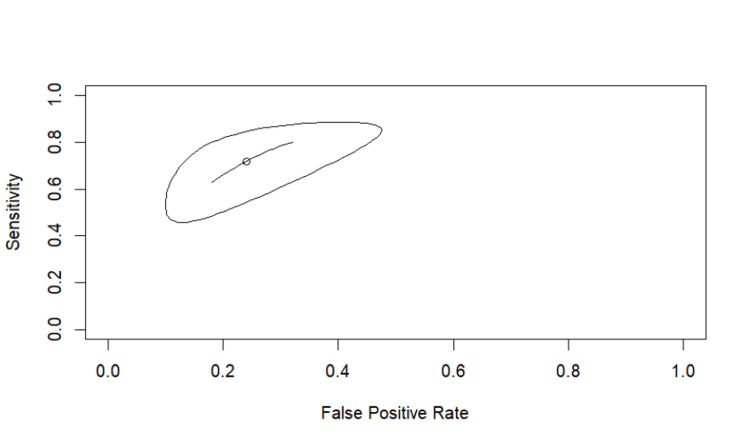
SROC curve for IVIM-D* The SROC curve for IVIM-D* was generated using Reitsma’s bivariate random-effects model. Studies shown are Qiu et al. [16] and Yu et al. [17]. IVIM, intravoxel incoherent motion; D*, pseudodiffusion coefficient; SROC, summary receiver operating characteristic

Two studies reported diagnostic accuracy data for D*, enabling bivariate SROC analysis. IVIM-D* demonstrated moderate diagnostic performance, with a pooled sensitivity of 0.718 (95% CI: 0.512-0.861) and a FP rate of 0.240 (specificity≈0.760, 95% CI for FPR: 0.120-0.424). The summary AUC was 0.803, indicating lower overall accuracy compared with IVIM-D. Heterogeneity was minimal across all indices (I²≈0%), suggesting consistent findings between the two included studies. The corresponding SROC curve is shown in Figure 6.

Comparative Performance: Overlay SROC

**Figure 7:**
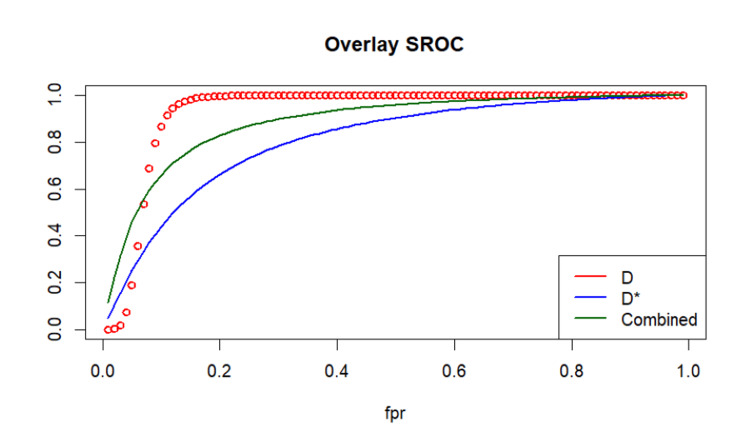
Overlay SROC comparing D, D*, and combined parameters Overlay plot displaying SROC curves for IVIM-D (red), IVIM-D* (blue), and the combined advanced parameter model (green). Studies shown are Qiu et al. [16] and Yu et al. [17] IVIM, intravoxel incoherent motion; D*, pseudodiffusion coefficient; D, diffusion coefficient; SROC, summary receiver operating characteristic; fpr, false positive rate

Comparison of the bivariate SROC models demonstrated that IVIM-D outperformed IVIM-D* in overall diagnostic accuracy. IVIM-D achieved a higher summary AUC (0.927 vs. 0.803) and better pooled sensitivity (0.847 vs. 0.718) and specificity (0.903 vs. 0.760). IVIM-D also showed a more compact confidence region and steeper SROC profile, reflecting greater discriminative ability. In contrast, D* demonstrated wider uncertainty and a flatter curve, consistent with its more variable performance across studies. The comparative overlay plot is shown in Figure 7.

Discussion

This systematic review and meta-analysis evaluated the diagnostic accuracy of advanced diffusion MRI parameters, including IVIM-derived metrics (D, D*, or f), DTI measures (FA, AD, MD, or RD), and physiologic contrasts such as APTw and T1 mapping, compared with conventional ADC-based DWI for identifying LNM in colorectal cancer. Across the included studies, IVIM-D consistently demonstrated higher diagnostic performance than ADC, reflected by superior pooled sensitivity, specificity, and AUC values. The summary ROC curves generated in R further supported this trend, showing a clear separation of IVIM-D curves from ADC and perfusion-based parameters.

Our findings align with prior work demonstrating the superiority of IVIM-D over ADC. Qiu et al. [16] reported that D achieved an AUC of 0.946 compared with 0.897 for ADC in rectal cancer nodal staging. Similarly, Yu et al. [17] found higher accuracy for D (AUC 0.885) relative to other IVIM metrics. Additional studies by Sun et al. [19] and Li et al. [20] further support the reliability of D for characterizing rectal tumors and lymph nodes. The advantage of D likely stems from its ability to isolate true molecular diffusion, whereas ADC is influenced by perfusion effects, b-value selection, and noise [21].

Perfusion-related parameters, particularly D*, exhibited lower diagnostic reliability in our pooled analysis. This is consistent with technical evaluations by Wu et al. [22] and Meeus et al. [23], who reported high variability, low signal-to-noise ratio, and limited reproducibility of D* across scanners, acquisition protocols, and fitting algorithms. Given these limitations, D* should be interpreted cautiously and is unlikely to serve as a primary biomarker for nodal staging.

DTI parameters, including FA, AD, MD, and RD, also demonstrated promising diagnostic potential. In the study by Yamada et al. [15], FA and AD achieved AUCs of 0.976, underscoring their sensitivity to microstructural disruption associated with metastatic infiltration. Prior research similarly supports the role of DTI in detecting architectural changes in malignant nodes [24,25], indicating that DTI metrics may serve as useful complementary markers.

Additional physiologic contrasts such as APTw and T1 mapping also showed potential diagnostic value, as demonstrated by Wang et al. [12]. These findings align with emerging radiomics and machine-learning studies that suggest enhanced performance when multiple MRI sequences are integrated [26,27]. In our analysis, combined parameters showed improved accuracy over individual metrics, supporting ongoing movement toward multiparametric MRI assessment.

Accurate detection of metastatic lymph nodes remains essential for guiding neoadjuvant therapy, surgical planning, and organ-preservation strategies [28]. Given the limitations of size-based criteria and the inconsistent performance of ADC alone, incorporating IVIM-D, selected DTI metrics, and potentially multiparametric approaches may enhance preoperative confidence and reduce reliance on morphology.

This review has several strengths, including standardized forest plots, bivariate SROC modeling, QUADAS-2 assessment, and inclusion of both node-based and patient-level data. To our knowledge, it is the first synthesis to directly compare the SROC performance of D, D*, and combined metrics. However, several limitations should be acknowledged. The number of eligible studies was small, and some sample sizes were modest. MRI acquisition parameters varied considerably across studies, including field strength, b-values, model-fitting methods, and region of interest (ROI) placement, contributing to methodological heterogeneity. Histopathologic standards were not uniform, and blinding procedures were not consistently reported. These factors may influence generalizability.

Future research should focus on large, multicenter prospective studies using standardized IVIM and DTI acquisition protocols. Harmonization of IVIM fitting methods, particularly for D*, is especially needed. Integrating diffusion-based parameters with radiomics, artificial intelligence, and multiparametric MRI may further improve diagnostic performance. Establishing clinically applicable threshold values for D, DTI metrics, and combined models will be essential for routine implementation.

## Conclusions

This systematic review and meta-analysis demonstrate that advanced diffusion MRI, particularly the IVIM-derived diffusion coefficient (D), offers meaningful improvements over conventional ADC for detecting LNM in colorectal cancer. IVIM-D consistently showed higher pooled sensitivity, specificity, and AUC, with minimal between-study heterogeneity, underscoring its robustness and potential clinical utility. In contrast, the perfusion-related parameter D* exhibited only moderate and more variable diagnostic performance. DTI metrics also showed promising accuracy, although evidence was limited to individual studies. Combined multiparametric approaches demonstrated improved performance in comparative SROC analysis, supporting further exploration of integrated diffusion and physiologic imaging strategies. Given the limitations of size-based criteria and conventional DWI, incorporating advanced diffusion parameters, especially IVIM-D, into preoperative MRI protocols may enhance diagnostic confidence and better inform treatment planning. Future multicenter studies with standardized acquisition protocols, harmonized IVIM/DTI fitting methods, and larger patient cohorts are needed to validate these findings and support their routine implementation in clinical practice.
